# Editorial: Consequences of the COVID-19 pandemic for evidence-based public health measures fostering child and adolescent mental health

**DOI:** 10.3389/fpubh.2024.1470043

**Published:** 2024-08-16

**Authors:** Stephan Bender

**Affiliations:** Department of Child and Adolescent Psychiatry, Psychosomatics and Psychotherapy, Faculty of Medicine and University Hospital Cologne, University of Cologne, Cologne, Germany

**Keywords:** child, adolescent, public health, SARS-CoV-2, pandemic, surveillance, psychosomatic, mental health

The SARS-CoV2 pandemic has had a major impact on infants, children, and adolescents. While children and adolescents are most often only mildly affected by somatic symptoms of a SARS-Cov2 infection, significant challenges in this age group have been identified regarding mental health as a consequence of public health measures to protect adults and elderly people. These challenges were associated with social distancing and public health measures employed to limit infection rates. Different countries adopted various public health strategies concerning the extent of social isolation for children, such as the duration and manner of school closures. Pandemic isolation as a natural experiment allows for assessing the consequences for the psychosocial development and mental health of the next generation. In Germany, this endeavor was undertaken by the project coverCHILD, funded by the Ministry of Education and Science (BMBF), and some results of the project are illustrated in this Research Topic. However, most articles from this Research Topic span the entire world, highlighting the importance of intercultural comparisons and international cooperation during global crises.

In a systematic review, Orban et al. found that children and adolescents experienced heightened mental health problems, specifically internalizing symptoms like anxiety and depression. Further, there was a decline in their overall health-related quality of life over the course of the pandemic that did not necessarily subside when lockdowns ended. Therefore, the SARS-Cov2 pandemic had a well-documented long-lasting effect on the quality of life and dimensionally assessed mental health symptoms. As Loy, Klam et al. point out, a combined assessment of reports by caregivers and self-reports of adolescents is needed, as well as country-specific normative values, to obtain a full picture of the mental health status. With respect to the SDQ, subtle differences can be found even between Western European countries. Thus, it is even more important that there is compelling evidence around the globe for the effects of social distancing in various populations: College students in the Southeastern US showed elevated symptoms of anxiety and post-traumatic stress (Chenneville et al.) and Hoa et al. report higher rates of anxiety disorder symptoms in a Vietnamese school student population when the Hanoi government introduced an extended period of social distancing. Comparing data between countries with strict and less strict public health measures seems especially valuable to distinguish general effects (climate change, etc.) from pandemic effects, despite all caveats about country-specific confounding factors. In Kuwait (Alamiri et al.), elevated rates of categorical anxiety disorders were described, with lockdown duration and sex of the child being important predictors for mental disorders. Therefore, both analyses across different countries as well as over different periods in the same country point toward a dose-effect with respect to the social isolation of children. Vulnerable groups were especially affected by pandemic measures, such as families with reduced income during the pandemic, and children with chronic disease or disability (Garcia de Avila et al.), as shown in this Research Topic in a Brazilian sample. These findings fit well into the worldwide literature indicating that more effective targeted support for vulnerable groups will be a challenge for the next pandemic. In adolescents with symptoms of depression, the prevalence of non-suicidal self-injury increased in China compared to pre-pandemic levels, pointing toward increased disease severity in this group (Hu et al.).

Not only did affective and anxiety disorders increase, but also the hospital admission rates for eating disorders went up in Germany (Silber et al.). In line with this finding, psychosomatic complaints such as sleeping problems and disorders have increased. A relationship between pandemic healthcare measures is suggested by the association of sleep quality with gender, limited time spent on outdoor activity, and prolonged electronic entertainment time (Ji et al.). Sleep problems, in turn, can have broader and secondary effects on mental health, as daytime dysfunction is related to sleep problems (Ji et al.). Moreover, Zou et al. show in the Research Topic that hostility mediates the relationship between sleep disturbances and aggression, i.e., sleep disturbances tend to increase aggression, especially in hostile people. Thus, the general effects of social deprivation and lack of sports and outdoor activities can affect circadian rhythms and indirectly a broad spectrum of mental disorders in addition to direct effects on anxiety or mood. In times of digitalization of medical routine care data, ways to rapidly aggregate and evaluate standardized mental health assessments along the lifespan will help to close the empty spaces in our knowledge and to rapidly assess the effects of any crisis as well as the public health measures to face it.

So far, the public health measures and family support applied during the SARS-Cov2 pandemic were not able to sufficiently alleviate parent stress. The most predictive factor for high parenting stress at follow-up in a German (Bavarian) sample of parents of toddlers was high parenting stress at baseline (Buechel et al.). This was also true for parental affective symptoms (depression/anxiety) and child mental health problems. Parents remained burdened. This burden was not limited to parents but also affected professional primary healthcare workers in the post-pandemic era in China (Liu et al.): Female gender, being divorced or widowed, being a nurse, years of working experience, working seniority, monthly income, and experience of workplace violence were identified as associated factors. Targeted interventions are needed in the future to reduce depression and improve primary healthcare workers' wellness and mental health.

At the same time, public health measures to ameliorate the negative consequences of pandemic hygiene measures need to be derived from our data and experience in the past pandemic, despite the scientific need not to infer causality from correlations. Anxiety and post-traumatic stress symptoms were reduced in college students who exercised daily, pointing to the importance of sports and the possibility of being active (Chenneville et al.). On the other hand, the pandemic accelerated the development of telemedical approaches in child and adolescent mental health. Apart from video-conference-administered psychotherapy, Wüllner et al. review the availability of (mobile phone) apps and evidence about their effectiveness. However, they conclude that although the majority of studies favor the intervention relying on or including the app over the control group, most studies have low or very low quality. Further studies are needed to improve both mental health care and low-threshold interventions and recommendations under conditions of social distancing.

It has become clear that timely research is necessary to guide healthcare and welfare policies to provide adequate vaccination and surveillance strategies for children and adolescents to maximize safe social contact in the pandemic context. Therefore, the willingness of parents, as well as children and adolescents, to accept surveillance (testing for infections, e.g., in schools) and/or vaccination is crucial.

In Ethiopia, a lack of antenatal care follow-up, postponement of the vaccination schedule in the past, mothers with parity of greater than four, and poor knowledge of the mothers about immunization were identified as determinants of immunization defaulting (Masebo et al.). This implies that the educational background, psychosocial burden, health literacy, as well as general attitudes toward public health measures and vaccination, affect parents' adherence to recommended vaccination schedules.

In Germany, the acceptance of an easier SARS-Cov2 testing method, a PCR-based “lollipop” test (taking a swab into the mouth so it can soak with saliva), was consistently rated more acceptable than nasal swab antigen rapid tests in school surveillance (Loy, Kimmig et al.). Parents highly appreciated that the test yielded more reliable results than the antigen rapid tests. Children said that it felt less uncomfortable. However, it also became clear that regardless of the test method, subjects who did not favor SARS-Cov2 vaccination also tended to rate school surveillance poorly.

In sum, this Research Topic shows how both the quality of life and the mental and psychosomatic health of children and adolescents have been affected by the SARS-Cov2 pandemic and social distancing measures. It illustrates some psychopathological mechanisms and risk factors for these effects. Some data for future health policies with respect to surveillance measures, vaccination campaigns, physical activity, as well as digitally supported healthcare, are provided. We hope that this research will help the responsible politicians draw adequate conclusions and weigh the positive and negative effects of public health care measures for children in order to maximize their well-being during future crises.

